# Effects of soil and climatic factors on the potential distribution of *Castanopsis eyrei* in China

**DOI:** 10.3389/fpls.2026.1763981

**Published:** 2026-02-25

**Authors:** Jingjing Cao, Huipeng Yang, Yutong Xia, Shixin Zhang, Yao Li, Yanming Fang

**Affiliations:** Co-Innovation Center for Sustainable Forestry in Southern China, College of Life Sciences, Nanjing Forestry University, Nanjing, China

**Keywords:** base saturation, climate change, MaxEnt, precipitation, species distribution

## Abstract

The geographical distributions of plant species are being actively reshaped by climate change. *Castanopsis eyrei*, a cornerstone species of subtropical evergreen broad-leaved forests in China, plays a critical role in community assembly and carbon sequestration. Understanding the key factors driving shifts in its potential distribution is vital to maintain biodiversity and formulate effective conservation strategies. Here, by comparing the soil-topographic-bioclimatic model with the bioclimatic-only model, we found that soil (base saturation) and climate (annual mean temperature, precipitation of the coldest quarter) jointly constrain the potential distribution of *C. eyrei*. The bioclimate-only model predicted larger suitable areas, highlighting that non-climatic variables can substantially alter the potential distribution forecasts. For the period 2041–2060, both models projected relatively stable distributions under low-emission (SSP1-2.6) and high-emission (SSP5-8.5) scenarios, with the latter showing greater northward expansion likely associated with increased temperature and precipitation. The soil-topographic-bioclimatic models showed lower inter-scenario variability, suggesting that soil and topographic factors may buffer against the effects of climatic change within our modeling framework. Our study demonstrates the necessity of integrating non-climatic variables into species distribution models, and provides projections to guide future monitoring and conservation efforts for *C. eyrei*.

## 1 Introduction

Climate change is a critical driver of shifts in species geographical distribution (Cannon et al., 2018; Liao et al., 2019; Sun et al., 2025), posing potentially irreversible threats to global biodiversity. A meta-analysis further suggests that if global temperatures rise beyond 1.5 °C, species extinction rates will rise sharply, and under the highest emission scenario, approximately one-third of global species could face extinction threats (Urban, 2024). Therefore, elucidating how species distributions respond to climate change is fundamental for informing targeted conservation strategies, which are crucial for safeguarding biodiversity and managing natural resources.

Species distribution models (SDMs) are key tools for assessing spatiotemporal dynamics under global change, with the maximum entropy (MaxEnt) model favored for its computational efficiency and robust predictive performance (Pillet et al., 2022; Daru and Rock, 2023; Mi et al., 2023; Wang et al., 2023; Luo et al., 2025). Mounting evidence links species migration and distribution shifts to climate change, with higher latitudes and altitudes increasingly suitable habitats (Koo et al., 2015; Zhao et al., 2021; Wang et al., 2023; Zhu et al., 2025). Notably, these shifts are not unidirectional but vary with species adaptability: adaptable ones expand (Tian et al., 2023), while those with poor adaptability and a reliance on stable niches contract (Sigdel et al., 2024).

Fagaceae species, widely distributed across the country (Zhu et al., 2025), are ideal for studying climate-driven distribution shifts due to their broad environmental tolerances and resilience (Cannon et al., 2018; Alonso-Forn et al., 2021; Zhou et al., 2022; Schwörer et al., 2024). There has been mounting evidence that their distribution is influenced by climate change. However, these SDM studies on Fagaceae species have largely relied on bioclimatic variables alone (Li et al., 2016, Li et al., 2018; Ramírez-Preciado et al., 2019; Zhu et al., 2025), neglecting soil and topographic heterogeneity—a critical knowledge gap that may lead to biased projections of suitable habitats and undermine conservation planning. We selected *Castanopsis eyrei* (Champ. ex Benth.) Tutcher, a late-successional dominant species in Chinese subtropical evergreen broad-leaved forests (covering ~25% of China’s land area; Fan et al., 2016; Jing et al., 2022), to address this gap. This species occurs at 300 to 1700 m altitude (Shi et al., 2011; Zhou et al., 2022), modulates its nutrient-use strategy to adapt to soil nutrient fluctuations and plays key role in microhabitat provision, degraded ecosystem restoration, community assembly and carbon sequestration (Hu et al., 2009; Li et al., 2015; Gong et al., 2023). Methodologically, *C. eyrei’*s specific adaptation to acidic, nutrient-poor soils via mycorrhizal associations makes it an ideal test case for addressing the methodological gap of neglecting soil-topographic variables in Fagaceae SDMs (Gao et al., 2016).

Based on previous physio-ecological studies and preliminary climatic data (Gao et al., 2016; Jing et al., 2022), we hypothesize that: (1) the distribution of *C. eyrei* is expected to be strongly associated with the precipitation of the coldest quarter, annual mean temperature and soil base saturation. (2) The inclusion of soil-topographic variables will lead to significantly different projected distribution areas compared to climate-only models.

## 2 Materials and methods

### 2.1 Current species collection

**Figure 1:**
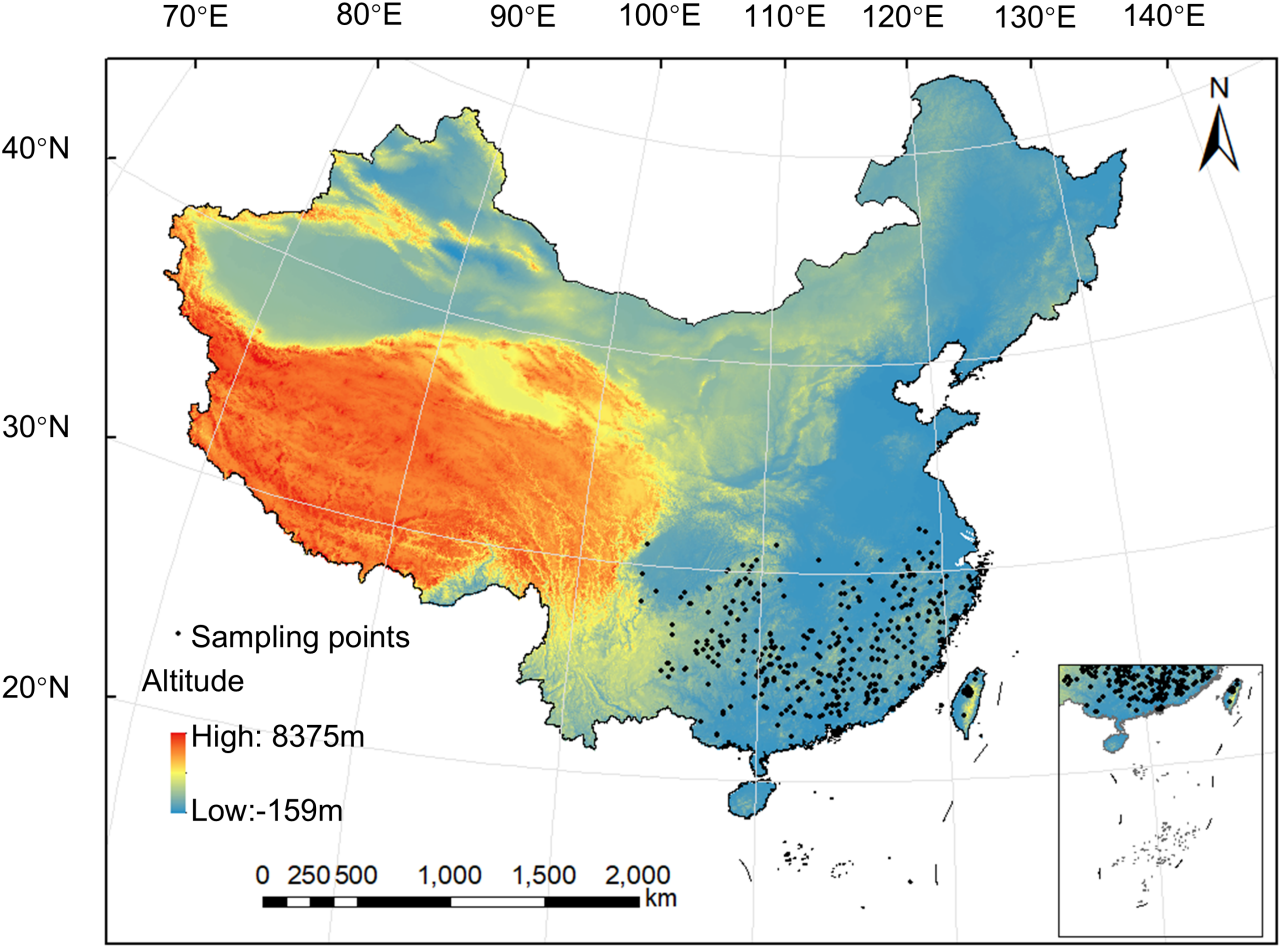
Current distribution records of *Castanopsis eyrei* (*n* = 423).

Current distribution data of *C. eyrei*, including 2,558 occurrence points, were retrieved from the Global Biodiversity Information Facility (GBIF) (https://www.gbif.org) and the China Virtual Herbarium (CVH) (https://www.cvh.ac.cn) (Wang et al., 2023). To address spatial bias in GBIF data, an ecologically constrained background selection was implemented. Considering that *C. eyrei* is a dominant species in the subtropical evergreen broad-leaved forests of China, we defined the background region as the subtropical evergreen broad-leaved forest zone in China (Zhu and Tan, 2024), and 10,000 background points were randomly sampled within this region to ensure ecological relevance to *C. eyrei* (Phillips et al., 2009; Merow et al., 2013). The study area was then delineated by generating a minimum bounding rectangle around the background point distribution. To mitigate edge effects, the rectangle was expanded by 5 pixels on all sides, resulting in the final research extent used for analysis. Occurrence data processing was performed as follows: Firstly, records lacking geographical coordinates (longitude or latitude), duplicate entries with redundant geographical information, and abnormal records were excluded from the original dataset. Spatial thinning was then conducted with a 1 km threshold (retaining only one data point per 1 km grid) to mitigate clustering effects and reduce spatial autocorrelation in model predictions, considering that the spatial resolution of the selected bioclimatic and soil variables was 30” (≈1 km) (Wang et al., 2023). Following the above filtering steps, 423 distribution points of *C. eyrei* were finally obtained (**Figure 1**). Consistent with the requirements of the MaxEnt model, these distribution records were formatted into a CSV file with columns for species name, longitude, and latitude.

### 2.2 Environmental variables for model simulation

Climate factors—particularly the 19 bioclimatic variables retrieved from WorldClim(https://worldclim.org/data/index.html)—are the most critical environmental variables and have been widely applied in species distribution prediction (Wang et al., 2023; Li et al., 2024; Zhu et al., 2025). Therefore, this study incorporated these bioclimatic factors to construct a bioclimatic-only model. Additionally, topographic and soil factors were added to establish a soil-topographic-bioclimatic model. Topographic variables (slope, aspect and elevation) were extracted from digital elevation model (DEM) data, while 20 soil variables were retrieved from the Harmonized World Soil Database (HWSDv.2.0). A total of 42 environmental variables (19 bioclimatic, 3 topographic and 20 soil variables) were compiled (**Supplementary Table 1**). All raster-format environmental variables were clipped and resampled using ArcGIS 10.8.

To optimize the establishment of the MaxEnt model and avoid multicollinearity (which can distort variable importance estimates and model predictions), principal component analysis (PCA) and Pearson correlation analysis were performed on all environmental variables to extract principal components with eigenvalues >1 (Tourne et al., 2019). A total of six such principal components were obtained for subsequent correlation analysis. When the absolute value of the correlation coefficient (|r|) between any two variables >0.8, the variable with weaker ecological significance was eliminated (Nan et al., 2024).

Notably, the 20 initially considered soil variables exhibited pronounced intercorrelations (|r| > 0.8 for all pairs, **Supplementary Figure 1**), indicating high redundancy and necessitating the selection of a single representative variable. We chose base saturation (BSAT) for its direct ecological relevance to *C. eyrei*: it governs soil fertility, pH buffering, and nutrient availability—critical factors for the species’ adaptation to acidic, nutrient-poor soils (Huang and Xu, 2010; Gao et al., 2016). Other soil variables were excluded due to collinearity with BSAT or lesser physiological relevance. The single exception was reference bulk density (REF_BULK), which was excluded primarily due to extensive data gaps. Importantly, none of the soil variables were strongly correlated with the bioclimatic variables (|r| < 0.6, **Supplementary Figure 1**), confirming soil as an independent environmental dimension in our models.

Finally, 7 environmental variables were selected for fitting the soil-topographic-bioclimatic model, comprising four bioclimatic variables, one soil variable, and two topographic variables (**Table 1**). Notably, these 4 bioclimatic variables were also employed for fitting the bioclimatic-only model.

**Table 1:** Information of 7 environmental variables used for fitting the soil-topographic-bioclimatic model.

Category	Environmental variable	Abbreviation	Unit
Soil variable	Base Saturation	BSAT	%
Bioclimatic variable	Annual Mean Temperature	BIO1	°C
	Mean Diurnal Range	BIO2	°C
	Precipitation Seasonality	BIO15	%
	Precipitation of Coldest Quarter	BIO19	mm
Topographic variable	Slope	SLOP	°
	Aspect	ASPE	°

### 2.3 MaxEnt model construction and evaluation

We constructed MaxEnt models using the 423 occurrence points, 10,000 background points, and the two sets of environmental variables (7 for the comprehensive model, 4 for the climate-only model). To ensure robustness and avoid overfitting, we systematically optimized model parameters before final predictions.

Parameter Optimization and Validation (Muscarella et al., 2014): We tested 40 combinations of feature classes (L, LQ, H, LQH, LQHP) and regularization multipliers (0.5 to 4 in steps of 0.5). The model with the lowest corrected Akaike Information Criterion (AICc) was selected, yielding optimal parameters of feature class = “L” and regularization multiplier = 0.5. To evaluate model performance and generalizability, we implemented a 2×2 spatial block cross-validation, partitioning the study area into four blocks to ensure spatially independent testing. The mean test AUC from this cross-validation was 0.821 (training AUC: 0.902). The low 10% omission rate and the modest difference between training and test AUC (0.08) indicated that overfitting was well-controlled.

Final Model Settings and Performance Assessment: For the prediction, the 423 selected *C. eyrei* distribution points and 10000 background points were loaded into MaxEnt (version 3.4.4) together with two sets of environmental variables—7 variables for constructing the soil-topographic-bioclimatic model and 4 variables for the bioclimatic-only model. We used a logistic output format, allocated 75% of occurrences for training and 25% for testing, and employed the bootstrap method with 10 replicates and a maximum of 1000 iterations (Phillips and Dudík, 2008; Warren and Seifert, 2011; Dormann et al., 2013). Model discriminative ability was assessed using the area under the receiver operating characteristic curve (AUC) (Phillips et al., 2006). AUC values were interpreted using standard ecological thresholds: 0.5–0.6 (poor), 0.6–0.7 (moderate), 0.7–0.8 (relatively high), 0.8–0.9 (high), and 0.9–1.0 (excellent) (Luo et al., 2025).

### 2.4 Evaluation of the importance of environmental variables

The importance of environmental variables in constraining the current geographical distribution pattern of *C. eyrei* was evaluated by percent contribution, permutation importance values, and Jackknife tests. Percent contribution reflects the proportion of the environmental variables’ contribution to the model’s gain. Permutation importance is quantified by randomly permuting variable values to assess the magnitude of subsequent model performance degradation. The Jackknife test evaluates variable impact on model performance by either including each variable individually or excluding it during modeling. These three approaches are commonly integrated to assess the importance of environmental variables in shaping species distribution.

### 2.5 Classification and future changes of the potential habitat

To project future distributions, we utilized bioclimatic data from three CMIP6 global climate models (BCC-CSM2-MR, MRI-ESM2-0, EC-Earth3-Veg) under two shared socioeconomic pathways (SSP1-2.6 and SSP5-8.5) for the period 2041–2060 (Wu et al., 2019; Yukimoto et al., 2019; Döscher et al., 2022). SSP1-2.6 represents a sustainable development pathway with low radiative forcing (2.6 W/m² by 2100), whereas SSP5-8.5 represents a high-emission pathway with strong radiative forcing (8.5 W/m² by 2100) (Tebaldi et al., 2021). Future bioclimatic variable data under these scenarios were used to project the fitted MaxEnt model, and the results were averaged to derive the potential distributions for *C. eyrei* for the 2041 to 2060 period under SSP1-2.6 and SSP5-8.5 scenarios. A critical methodological note is that soil and topographic variables were held constant in future projections, consistent with common practice but representing a simplification of potential long-term soil-climate feedbacks.

The continuous habitat suitability outputs (probability from 0 to 1) from both the comprehensive and climate-only models were classified into four suitability levels using Jenks’ natural breaks method (Jenks, 1967): unsuitable (0–0.10), low suitability (0.10–0.27), moderate suitability (0.27–0.54), and high suitability (0.54–1.00). For change analysis, a binary habitat map was created by reclassifying pixels with suitability < 0.10 as “unsuitable” and all others as “suitable”.

Changes in potential habitat between current and future periods were analyzed by overlaying the corresponding binary maps. Each pixel was assigned to one of four change categories: stable unsuitable, habitat loss (suitable to unsuitable), habitat gain (unsuitable to suitable), and stable suitable. Net change in the total suitable area was calculated as (future suitable area – current suitable area), with positive and negative values indicating expansion and contraction, respectively.

## 3 Results

### 3.1 Current model evaluations and critical environmental variables

**Figure 2:**
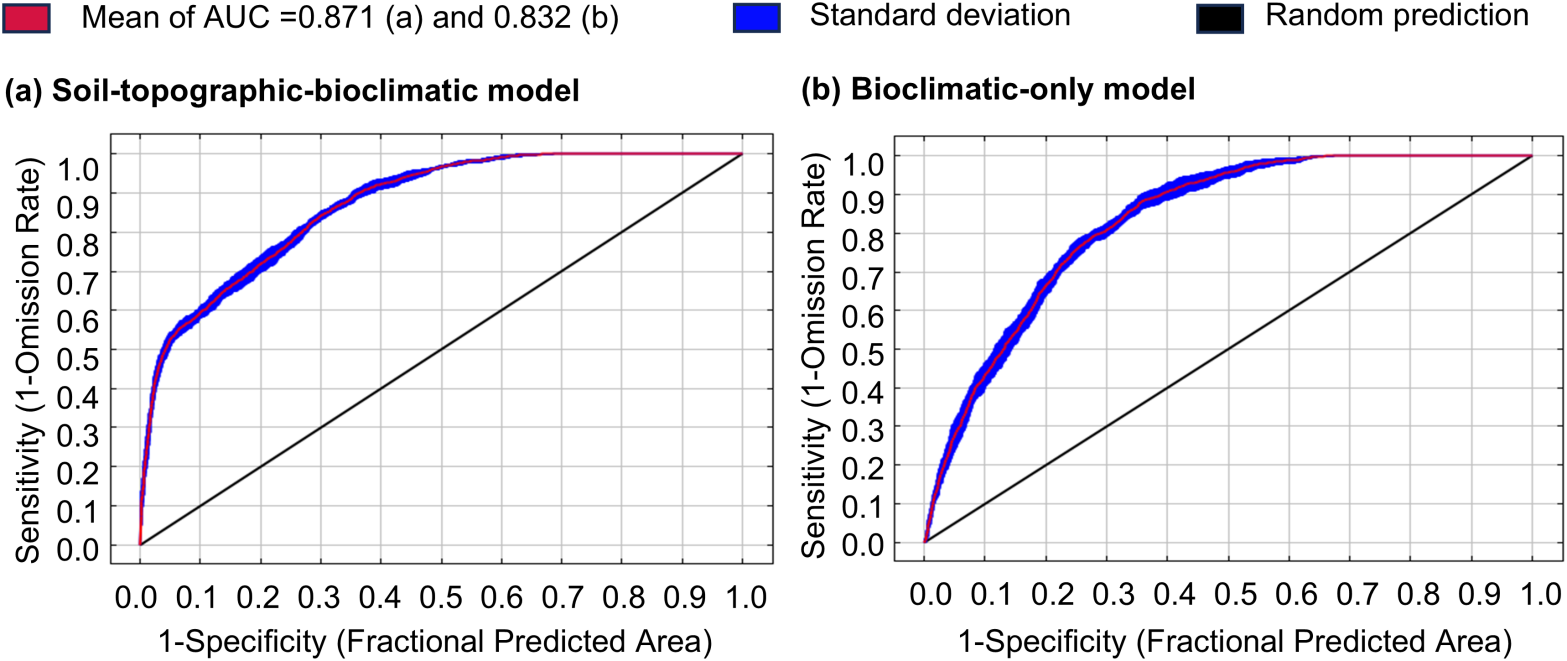
Reliability test of the MaxEnt model created for *Castanopsis eyrei* in **(a)** soil-topographic-bioclimatic model and **(b)** bioclimatic-only model.

The average test AUC values (± SD) of the soil-topographic-bioclimatic model and the bioclimatic-only model were 0.871 (± 0.005) and 0.832 (± 0.009), respectively, suggesting that the prediction results of the two current model in this study are highly accurate and reliable (**Figure 2**).

In the soil-topographic-bioclimatic model, the top three variables by percent contribution account for a cumulative importance of 93.60%: base saturation (BSAT, 66.20%), precipitation of coldest quarter (BIO19, 17.30%), annual mean temperature (BIO1, 10.10%). For permutation importance, the leading two variables are BSAT (53.90%) and BIO1 (32.80%), with a cumulative importance of 86.70% (**Table 2**). The bioclimatic-only model similarly identified BIO1 and BIO19 as dominant variables(**Supplementary Table 2**).

**Table 2:** Contribution of variables in the soil-topographic-bioclimatic model.

Environment variable	Abbreviation	Percent contribution (%)	Permutation importance (%)
Base Saturation	BSAT	66.20	53.90
Precipitation of Coldest Quarter	BIO19	17.30	4.70
Annual Mean Temperature	BIO1	10.10	32.80
Precipitation Seasonality	BIO15	3.00	7.80
Slope	SLOP	2.70	0.60
Mean Diurnal Range	BIO2	0.50	0.00
Aspect	ASPE	0.20	0.20

**Figure 3:**
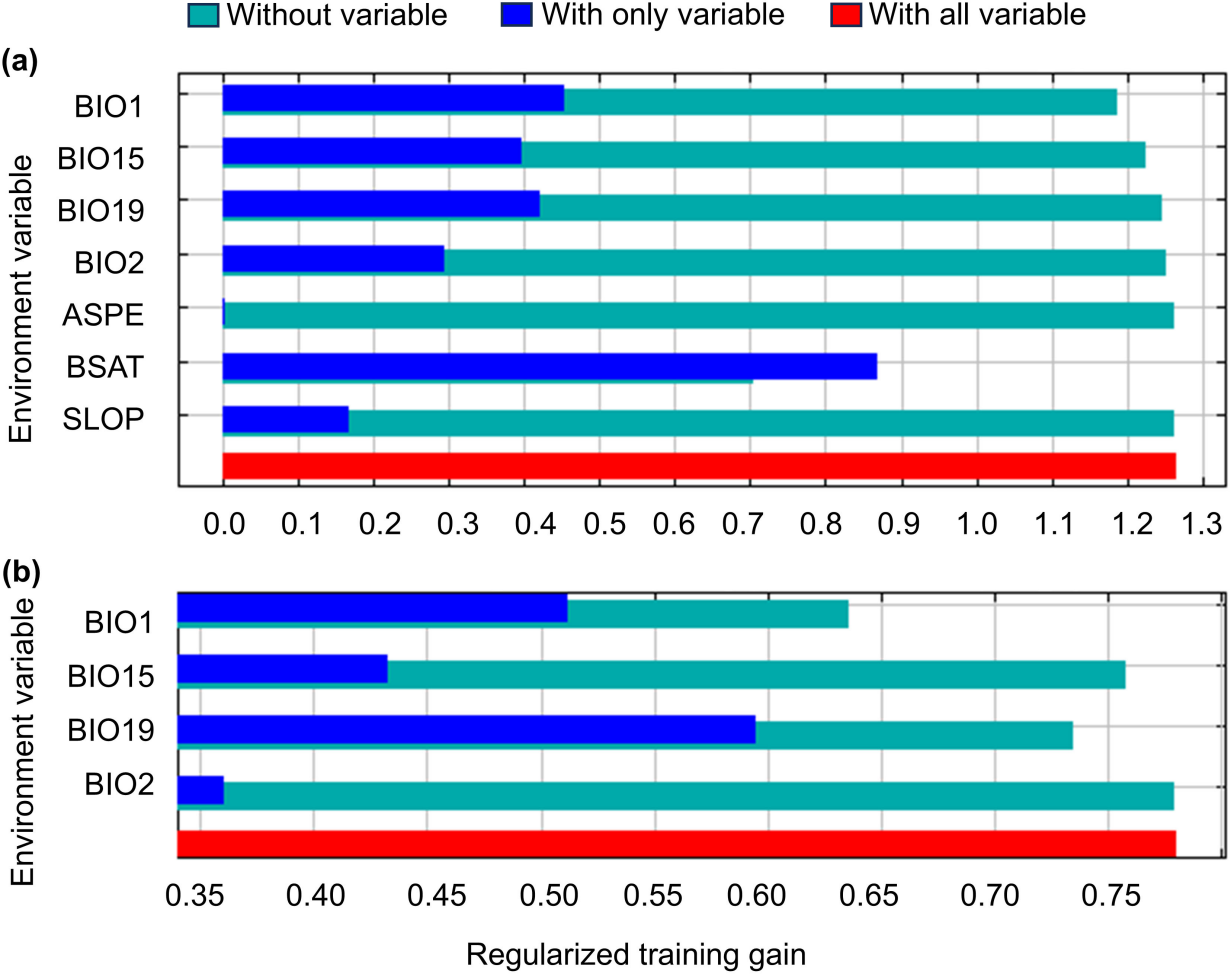
Jackknife test of variable importance for *Castanopsis eyrei* in **(a)** soil-topographic-bioclimatic model and **(b)** bioclimatic-only model.

Furthermore, the environmental variable importance assessment via Jackknife test reveals that simulating with individual variables yields the highest gain for BSAT (**Figure 3a**) and BIO19 (**Figure 3b**), suggesting it inherently contains the most meaningful ecological information (**Figure 3**).

Overall, based on the results presented above (**Table 2**; **Figure 3**), we identified three key environmental variables influencing the current distribution of *C. eyrei*: two climate-related variable (BIO19 and BIO1) and one soil variable (BSAT).

**Figure 4:**
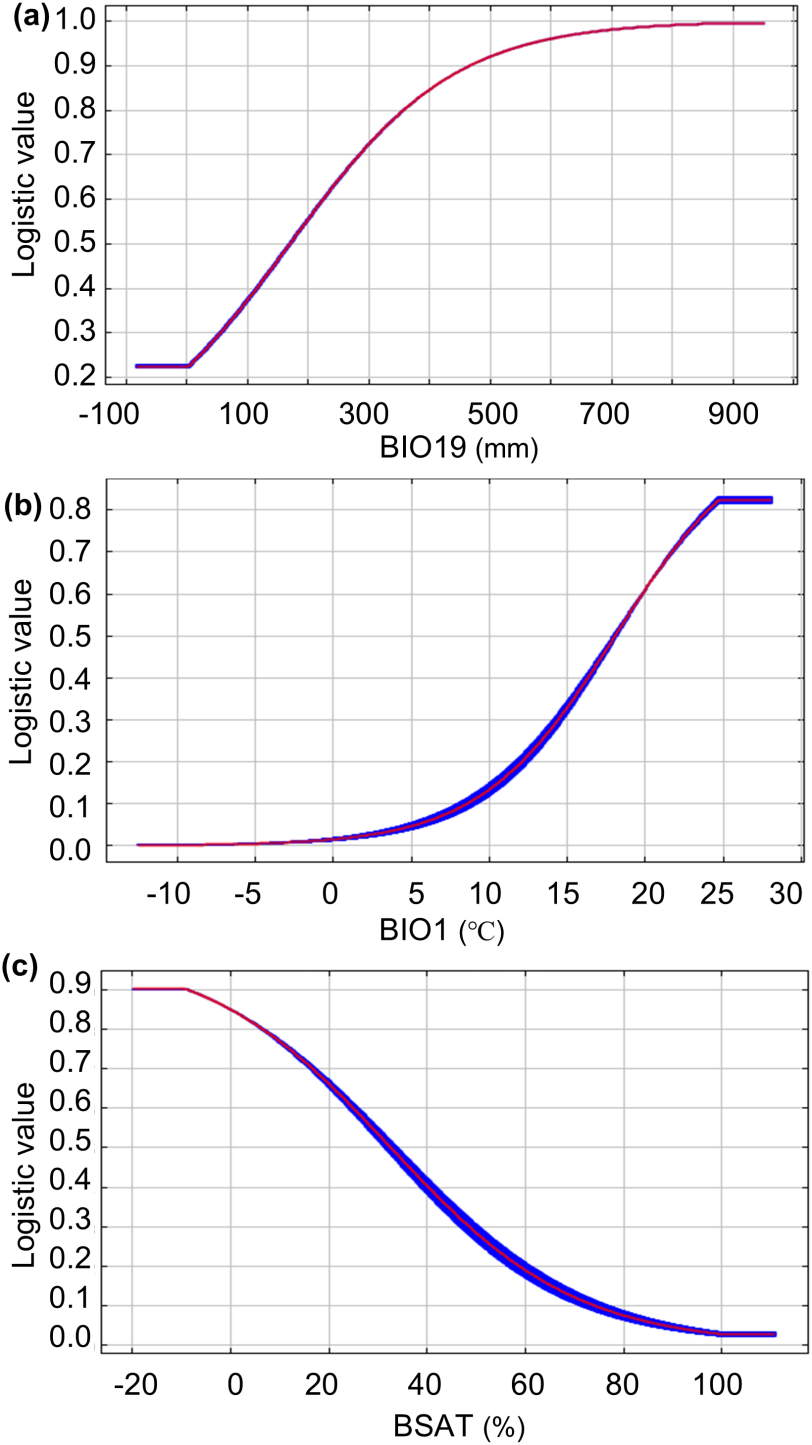
Response curves of major environmental variables for the probability of presence of *Castanopsis eyrei*, **(a)** precipitation of coldest quarter (BIO19), **(b)** annual mean temperature (BIO1) and **(c)** base saturation (BSAT).

The response curves of these key variables in the soil-topographic-bioclimatic model are shown in **Figure 4**. Analysis revealed distinct patterns: the probability of suitable habitat increased and then stabilized with rising BIO19 and BIO1, whereas it showed a declining trend with increasing BSAT. The approximate suitable ranges, derived from the response curves, were: BIO19: 187.84–947.00 mm; BIO1: 17.87–27.69 °C; and BSAT: 0–24.81%.

### 3.2 Prediction of *C. eyrei* current and future potential distribution

**Figure 5:**
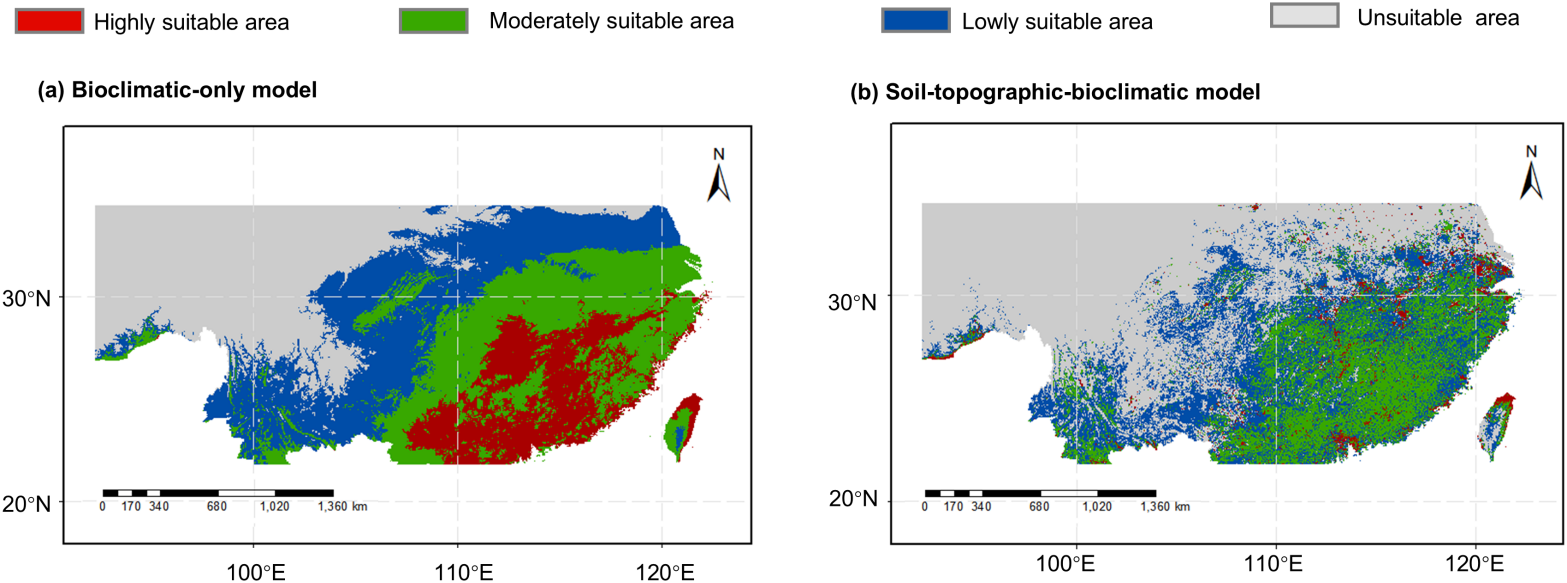
Current potential suitable areas for *Castanopsis eyrei*. Models driven by **(a)** only bioclimatic variables; **(b)** bioclimatic, soil and topographic variables.

The projected current distributions of *C. eyrei* from the two models are presented in **Figure 5**. Both models indicate that the primary suitable habitats located in southern China. The bioclimatic-only model projected a larger total and highly suitable area. However, the overall geographical range of suitability was broadly consistent between models, encompassing provinces and regions including Sichuan, Guizhou, Yunnan, Guangxi, Hunan, Hubei, Henan, Anhui, Jiangsu, Jiangxi, Zhejiang, Fujian, Guangdong, Taiwan, Chongqing, Shanghai, and Xizang. Compared to the bioclimatic-only model, the soil-topographic-bioclimatic model yielded a more fragmented pattern of suitable habitat.

**Figure 6:**
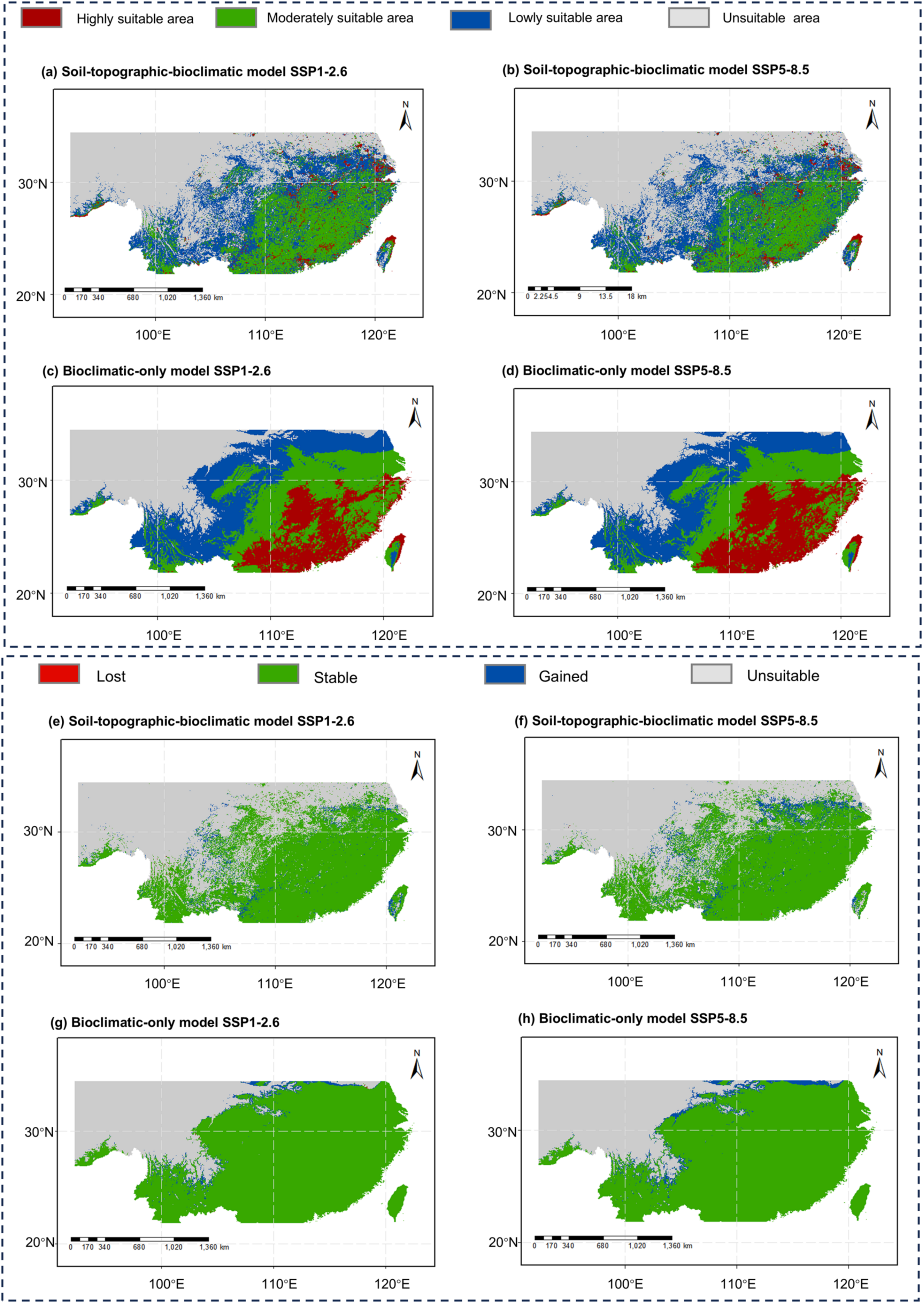
Average potential suitable areas **(a-d)** and Changes in the average potential geographical distribution **(e-h)** of *Castanopsis eyrei* under SSP1-2.6 [**(a, c, e, g)** low-emission scenario] and SSP5-8.5 [**(b, d, f, h)** high-emission scenario] scenarios. Models driven by **(a, b, e, f)** bioclimatic, soil and topographic variables; **(c, d, g, h)** only bioclimatic variables.

For the future period 2041–2060, ensemble projections (averaged across three CMIP6 climate models) under both SSP1-2.6 and SSP5-8.5 scenarios indicate a relatively stable distribution for *C. eyrei* (**Figures 6a–d**). Consistent with current patterns, the bioclimatic-only model again projected larger total and highly suitable areas than the comprehensive model under both future scenarios.

### 3.3 The future changes of the potential distribution area

Both models projected a relatively stable core distribution for *C. eyrei* by 2041–2060, with dynamic changes (primarily expansion) occurring sporadically along the northern and western margins of its current range (**Figures 6e–h**).

Comparison of the two future scenarios within each model revealed that the soil-topographic-bioclimatic model exhibited reduced inter-scenario variability in the spatial pattern of change, whereas the differences between SSP1-2.6 and SSP5-8.5 were more pronounced in the bioclimatic-only model.

Comparing the two models under the same scenario, a clear contrast emerged: the bioclimatic-only model projected more spatially aggregated and contiguous areas of change. In contrast, the soil-topographic-bioclimatic model yielded a more fragmented pattern of change, with patches of expansion interspersed along the range margins.

## 4 Discussion

### 4.1 Evaluation of the MaxEnt model in the predicting species potential distribution

To assess the role of multi-environmental integration in species distribution modeling, we compared models based solely on bioclimatic variables with those incorporating additional factors such as soil properties and topography. Differences in key predictors and the projected future changes in suitable habitat areas for *C. eyrei* were analyzed. Although, model accuracy and reliability were further strengthened through parameter optimization and spatial cross-validation, building on the use of multi-environmental variables and climate system averaging. Several key limitations should be noted.

First, this study did not account for interspecific interactions or human disturbance (Sanczuk et al., 2022; Mou et al., 2025) which may lead to deviations between predicted and actual distributions. Despite robust model performance (AUC > 0.8), results should be interpreted cautiously. Biotic interactions (e.g., competition) can constrain the realized niche, leading to overestimated suitability where it is excluded by other species (Wisz et al., 2013; Dormann et al., 2018). Additionally, anthropogenic disturbances such as land-use change—not fully captured in the model—can further restrict habitat and dispersal (Gallardo et al., 2015). Thus, the actual distribution may be more limited than the climatically suitable areas predicted. Second, as is common in species distribution modeling, soil properties were treated as static under future climate scenarios—a pragmatic but ecologically unrealistic simplification that overlooks climate-driven soil processes (Lehmann and Kleber, 2015; Ni and Vellend, 2023; Wang et al., 2023). These omissions may affect projected habitat suitability and add uncertainty to long-term predictions. Future modeling efforts should therefore aim to integrate a wider range of static and dynamic biotic and abiotic factors (Stanton et al., 2011).

Comparison between model predictions and actual distribution records of *C. eyrei* shows both consistencies and discrepancies. The MaxEnt model indicated that the species is currently distributed mainly in southeastern China, south of the Qinling Mountain-Huaihe River Line and east of the Tanaka-Kaiyong Line (**Figure 5**), which generally matched known records (**Figure 1**). However, notable. The model also suggested scattered suitable areas west of the Tanaka-Kaiyong Line (e.g., Yunnan and Xizang), and in Henan provinces, where the species has not been documented. These mismatches may arise because the model output reflects distribution probability rather than realized presence (Liu et al., 2021), and may be further influenced by uncertainties in CMIP6 climate projections (e.g., in warming rates and precipitation patterns; (Wild, 2020; Zhao et al., 2020; Wang et al., 2023). Given the lack of validating records, Henan, Yunnan, and Xizang are excluded from the subsequent discussion.

### 4.2 The key variables affecting the potential distribution of *C. eyrei*

As hypothesized, the distribution of *C. eyrei* is primarily constrained by BIO19, BIO1 and BSAT, which collectively depict a species that favors humid and warm environments and acidic soils. This aligns with studies identifying precipitation as key for *C. eyrei* (Ouyang et al., 2023). Furthermore, *C. eyrei*’s preference for acidic soils matches field observations (Gao et al., 2016). Together, this supports the modeled ‘high humidity–acidic soil’ niche as ecologically consistent.

BIO19 (187.84 to 947.00 mm), providing sufficient moisture for growth while avoiding frost damage from excess precipitation. Highly correlated precipitation variables—precipitation of driest month (BIO14), annual precipitation (BIO12) and precipitation of wettest quarter (BIO16) (with a correlation coefficient≥0.8; **Supplementary Figure 1**) —collectively define the species’ hydrological niche, indicating a preference for moderate moisture availability and tolerance to both drought and waterlogging.

BIO1 (17.87 to 27.69 °C) critically shapes the distribution of *C. eyrei* by imposing low-temperature stress that limits seed germination and hinders growth, thereby restricting northward expansion (Li et al., 2016). This thermal gradient underpins the species’ current and projected distribution, which is concentrated in southern China (south of the Qinling–Huaihe River Line). Future warming may reduce cold stress and facilitate a northward shift. However, it may also lead to insufficient chilling for vernalization and altered phenology, potentially disrupting ecological interactions with pollinators and seed dispersers and affecting reproduction (Parmesan and Yohe, 2003; Rafferty and Ives, 2011; Kudo and Ida, 2013; Taiz and Zeiger, 2015; Lang et al., 2025). Other temperature variables strongly correlated with BIO1 (BIO5, BIO6, BIO8–BIO11; **Supplementary Figure 1**) also significantly influence distribution. In summary, *C. eyrei* prefers warm climates, and its distribution is strongly temperature-dependent, aligning with observed poleward shifts under global warming.

BSAT, defined as the percentage of the soil’s cation exchange capacity occupied by exchangeable base cations, is a direct indicator of soil pH and nutrient availability. Low BSAT corresponds to acidic conditions, explaining the general geographical pattern: soils in arid and semi-arid northern regions typically have high BSAT and pH, while humid southern soil exhibit lower values. BSAT also serves as a key fertility metric soils are classified as highly fertile (BSAT ≥ 80%), moderately fertile (50-80%), or of low fertility (<50%) based on this metric (Huang and Xu, 2010). The identified BSAT threshold of 0–24.81% indicates that *C. eyrei* predominantly grows in acidic, low-fertility soils. This aligns with the properties of the predominant red and yellow soils in southern China (Wang et al., 2020), helping to explain the species’ confinement to regions south of the Qinling Mountain–Huaihe River Line.

Furthermore, a low BSAT threshold may as a critical factor defining *C. eyrei*’s suitable habitat. We hypothesize that this strong statistical association may be underpinned by a physiological tolerance to the acidic and infertile conditions that low BSAT signifies. This hypothesis, however, requires future validation through direct experimentation, such as measuring plant growth, nutrient uptake, or stress responses of *C. eyrei* across a controlled soil BSAT gradient in common garden or pot trials.

### 4.3 Changes in potential suitable area for *C. eyrei*

Floristic analysis indicates that *C. eyrei* is currently distributed mainly south of the Qinling–Huaihe River Line and east of the Tanaka–Kaiyong Line (Fan et al., 2013), with its core range in the subtropical regions south of the Yangtze River. This distribution aligns closely with the core of the China-Japan plant subregion (Wu et al., 2010), reflecting the strong dependence of *C. eyrei* within the humid monsoon climate and acidic soil characteristic of this floristic region.

First, we compared distribution patterns from the soil-topographic-bioclimatic model and the bioclimatic-only model. When only climatic were considered, predicted suitable habitat appeared more aggregated, with smooth patterns of range expansion—a result of the broad spatial gradients in macroclimatic variables that mask fine-scale habitat heterogeneity. In contrast, adding soil and topographic variables produced a more fragmented habitat structure, especially near the distribution margins, forming a “microhabitat mosaic” (Guisan and Thuiller, 2005). This pattern may align better with the actual ecological context of *C. eyrei*, capturing the effect of localized environmental variation.

Second, future projections suggest suitable range will remain largely stable, with only minor shifts along its northern and western margins. This relative stability within our modeling framework suggests that the projected climate changes may not exceed the fundamental climatic niche of *C. eyrei* as defined by the variables used. However, this interpretation must be tempered by the acknowledged limitations of our model, including the static treatment of soils and the exclusion of biotic interactions, which add substantial uncertainty to long-term predictions. Peripheral populations, existing near the species’ environmental tolerance limits (Guisan and Thuiller, 2005; Hampe and Petit, 2005), are likely more sensitive. Even modest climatic changes could therefore push local conditions beyond (or into) these thresholds, potentially causing range contraction or expansion depending on whether future conditions become unsuitable or newly favorable.

Third, we speculated that improved habitat conditions may facilitate localized expansion at the western and northern boundaries. In the west, complex topography creates a mosaic of microclimates, allowing certain refugial sites (e.g., shaded valleys, moist slopes) to maintain or even improve suitability for *C. eyrei* despite regional warming (Guisan and Thuiller, 2005). Such microrefugia can buffer the species against adverse climate impacts (Dobrowski, 2011). In the north, a slight increase in temperature and precipitation (especially during the coldest quarter) is projected to alleviate cold and water stress, facilitating limited expansion along the northern margin (Ding et al., 2024). These newly suitable habitats could act as ecological corridors, connecting otherwise fragmented forest patches and enhancing landscape connectivity—a key factor in maintaining gene flow and supporting biodiversity conservation under global change.

Finally, we compare distributional responses under different climate scenarios within the same model. In both models, the high-emission scenario (SSP5-8.5) projected greater northward expansion of the distribution boundary than the low-emission scenario (SSP1-2.6). The expansion may be attributed to a more pronounced increase in precipitation and temperature under SSP5-8.5, which could enhance habitat suitability in previously drier and cooler regions (Nazarenko et al., 2022). In contrast, the soil–topography–bioclimatic model showed less variability between scenarios, suggesting that soil properties and topographic complexity buffer against scenario-specific climatic differences (Cartwright et al., 2020; Trew and Maclean, 2021; McNichol et al., 2024). An additional factor contributing to the limited variability could be our treatment of soil properties as static—a methodological simplification that does not account for their potential long-term evolution under changing climates.

### 4.4 Future perspectives

Future research to improve the predictive power of species distribution models and to support the conservation of *C. eyrei* should focus on three key areas: model refinement, elucidation of acid-tolerance mechanisms, and the development of targeted conservation strategies.

First, model refinement. This study shows that including soil and topographic variables—beyond bioclimatic factors—reveals how local conditions buffer climate change impacts. Future models should integrate a broader range of biotic and abiotic variables, such as dispersal ability, phenological responses, interspecific competition, mycorrhizal associations (Bellard et al., 2012; Delavaux et al., 2019; Liao et al., 2019; Sanczuk et al., 2022; Peng et al., 2024), as well as anthropogenic pressures like land-use change and nitrogen deposition (Pillet et al., 2022; Sanczuk et al., 2024).

Secondly, elucidating acid-tolerance mechanisms. BSAT was a key predictor of *C. eyrei* distribution. We hypothesize that the species’ dominance in low-BSAT soils stems from a specialized acid-tolerance adaptation. Research should prioritize uncovering the underlying physiological and molecular bases—for instance, by identifying key acid-tolerance genes and screening for efficient mycorrhizal partners. These insights could guide ecological restoration in acid-degraded ecosystems and inform the breeding of acid-resistant crops.

Finally, developing conservation strategies. To enhance the resilience of *C. eyrei* under future climates, efforts could include breeding drought- or cold-tolerant varieties via hybridization or gene editing. Additionally, establishing ecological corridors within its current range would connect fragmented populations, promote gene flow, and improve long-term adaptive capacity (Gilbert-Norton et al., 2010; Qu et al., 2024).

## 5 Conclusions

This study employed the MaxEnt model to simulate the distribution of *C. eyrei*, a keystone evergreen broad-leaved species, under current and future climates. We identified BIO19, BIO1, and BSAT as the key environmental factors shaping its distribution, which is largely confined to the humid, warm and acidic-soil regions of southeastern China. Under future scenarios (2041–2060), the species’ overall distribution is projected to remain relatively stable, suggesting that the projected climate changes may not exceed its fundamental climatic niche as defined here. Potential limited expansion is projected along the northern and western margins, likely facilitated by increased temperature and precipitation as well as topographic microrefugia.

Methodologically, our comparative analysis suggests that incorporating soil and topographic variables can yield more fragmented and ecologically nuanced projections than climate-only models, highlighting the potential value of these factors for prediction. Consequently, we recommend that future SDM studies for similar species integrate these non-climatic factors. For conservation, the projected stability of the core range, alongside areas of potential change, offers a preliminary, spatially explicit framework that may aid in prioritizing long-term monitoring and in exploring strategies to maintain landscape connectivity under climate change.

## Data Availability

The original contributions presented in the study are included in the article/**Supplementary Material**. Further inquiries can be directed to the corresponding authors.

## Supplementary material

Supplementary Figure 1 Correlation heatmap of 42 environmental variables.

Supplementary Table 1 Information of 42 environmental variables.

Supplementary Table 2 Contribution of variables in bioclimatic-only model.
