# Supplementary figures and images for: Effects of soil and climatic factors on the potential distribution of Castanopsis eyrei in China
Source: Front Plant Sci. 2026 Feb 25;17:1763981. doi: 10.3389/fpls.2026.1763981 (PMC12975431; doi:10.3389/fpls.2026.1763981)

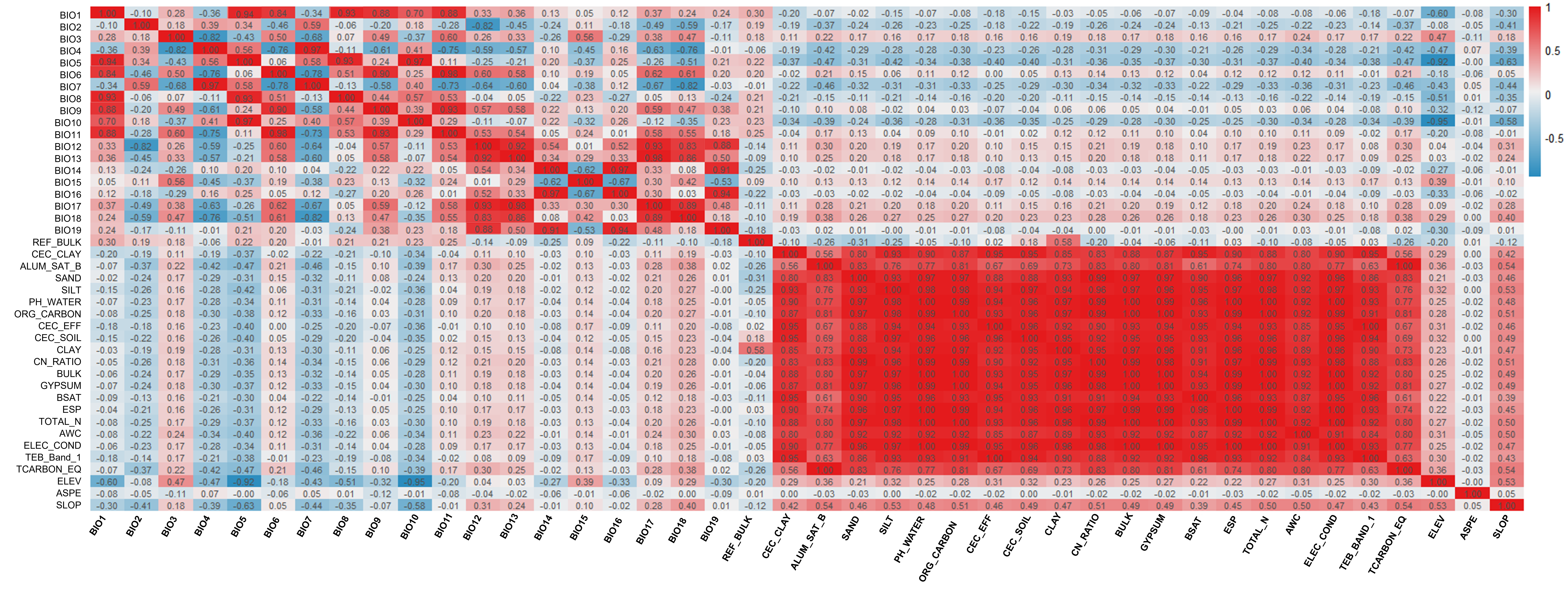

Supplement: Supplementary Figure 1 — Correlation heatmap of 42 environmental variables. [file Image1.tif]
