# Supplementary material for: Effects of soil and climatic factors on the potential distribution of Castanopsis eyrei in China
Source: Front Plant Sci. 2026 Feb 25;17:1763981. doi: 10.3389/fpls.2026.1763981 (PMC12975431; doi:10.3389/fpls.2026.1763981)
Supplement: Supplementary Table 1 — Information of 42 environmental variables. [file Table1.docx]

Table S1. Information of 42 environmental variables

|  | Environmental variable | Abbreviation | Unit |
| --- | --- | --- | --- |
| Soil variable | Reference Bulk Density | REF_BULK | g/cm³ |
|  | Cation Exchange Capacity of the Clay Fraction | CEC_CLAY | cmol(+)/kg clay |
|  | Exchangeable Aluminum Saturation | ALUM_SAT_B | % |
|  | Sand Content | SAND | % |
|  | Silt Content | SILT | % |
|  | pH in Water | PH_WATER | - |
|  | Organic Carbon Content | ORG_CARBON | % |
|  | Effective Cation Exchange Capacity | CEC_EFF | cmol(+)/kg soil |
|  | Cation Exchange Capacity of the Soil | CEC_SOIL | cmol(+)/kg soil |
|  | Clay Content | CLAY | % |
|  | Carbon to Nitrogen Ratio | CN_RATIO | - |
|  | Bulk Density | BULK | g/cm³ |
|  | Gypsum Content | GYPSUM | % |
|  | Base Saturation | BSAT | % |
|  | Exchangeable Sodium Percentage | ESP | % |
|  | Total Nitrogen Content | TOTAL_N | % |
|  | Available Water Capacity | AWC | mm/m |
|  | Electrical Conductivity | ELEC_COND | dS/m |
|  | Total Exchangeable Bases - Band 1 | TEB_BAND_1 | cmol(+)/kg soil |
|  | Total Carbon Equivalent | TCARBON_EQ | kg/m² |
| Bioclimatic variable | Annual Mean Temperature | BIO1 | °C |
|  | Mean Diurnal Range | BIO2 | °C |
|  | Isothermality | BIO3 | % |
|  | Temperature Seasonality | BIO4 | - |
|  | Max Temperature of Warmest Month | BIO5 | °C |
|  | Min Temperature of Coldest Month | BIO6 | °C |
|  | Temperature Annual Range | BIO7 | °C |
|  | Mean Temperature of Wettest Quarter | BIO8 | °C |
|  | Mean Temperature of Driest Quarter | BIO9 | °C |
|  | Mean Temperature of Warmest Quarter | BIO10 | °C |
|  | Mean Temperature of Coldest Quarter | BIO11 | °C |
|  | Annual Precipitation | BIO12 | mm |
|  | Precipitation of Wettest Month | BIO13 | mm |
|  | Precipitation of Driest Month | BIO14 | mm |
|  | Precipitation Seasonality | BIO15 | % |
|  | Precipitation of Wettest Quarter | BIO16 | mm |
|  | Precipitation of Driest Quarter | BIO17 | mm |
|  | Precipitation of Warmest Quarter | BIO18 | mm |
|  | Precipitation of Coldest Quarter | BIO19 | mm |
| Topographic variable | Elevation | ELEV | m |
|  | Slope | SLOP | ° |
|  | Aspect | ASPE | ° |
