# Supplementary material for: Effects of soil and climatic factors on the potential distribution of Castanopsis eyrei in China
Source: Front Plant Sci. 2026 Feb 25;17:1763981. doi: 10.3389/fpls.2026.1763981 (PMC12975431; doi:10.3389/fpls.2026.1763981)
Supplement: Supplementary Table 2 — Contribution of variables in bioclimatic-only model. [file Table2.docx]

Table S2. Contribution of variables in bioclimatic-only model

| Environment variable | Percent contribution (%) | Permutation importance (%) |
| --- | --- | --- |
| BIO19 | 60.80 | 20.70 |
| BIO1 | 36.00 | 65.00 |
| BIO15 | 3.00 | 13.20 |
| BIO2 | 0.20 | 1.00 |
